# Deep learning biomarker of chronometric and biological ischemic stroke lesion age from unenhanced CT

**DOI:** 10.1038/s41746-024-01325-z

**Published:** 2024-12-06

**Authors:** Adam Marcus, Grant Mair, Liang Chen, Charles Hallett, Claudia Ghezzou Cuervas-Mons, Dylan Roi, Daniel Rueckert, Paul Bentley

**Affiliations:** 1 Department of Brain Sciences, Imperial College London, London, UK; 2 Centre for Clinical Brain Sciences, University of Edinburgh, Edinburgh, UK; 3 Department of Computing, Imperial College London, London, UK; 4 Klinikum rechts der Isar, Technische Universität München, München, Germany

**Keywords:** Stroke, Stroke, Prognostic markers

## Abstract

Estimating progression of acute ischemic brain lesions – or biological lesion age - holds huge practical importance for hyperacute stroke management. The current best method for determining lesion age from non-contrast computerised tomography (NCCT), measures Relative Intensity (RI), termed Net Water Uptake (NWU). We optimised lesion age estimation from NCCT using a convolutional neural network – radiomics (CNN-R) model trained upon chronometric lesion age (Onset Time to Scan: OTS), while validating against chronometric and biological lesion age in external datasets (*N* = 1945). Coefficients of determination (R^2^) for OTS prediction, using CNN-R, and RI models were 0.58 and 0.32 respectively; while CNN-R estimated OTS showed stronger associations with ischemic core:penumbra ratio, than RI and chronometric, OTS (ρ^2^ = 0.37, 0.19, 0.11); and with early lesion expansion (regression coefficients >2x for CNN-R versus others) (all comparisons: *p* < 0.05). Concluding, deep-learning analytics of NCCT lesions is approximately twice as accurate as NWU for estimating chronometric and biological lesion ages.

## Introduction

Efficacy of acute ischemic stroke treatments depends, at the individual level, on biological lesion age: that is, where an acute lesion lies on the continuum between reversible ischemia and irreversible infarction. Biological age is closely related to chronometric lesion age – i.e. time from symptom onset – although these ages dissociate due to variability in tissue vulnerability and arterial collateral supply^1,2^. Established methods for estimating both biological and chronometric lesion age– MRI, or CT Perfusion (CTP) – introduce logistical hurdles, and delay treatments, compared to Non-Contrast CT (NCCT). Moreover, both imaging modalities are error-prone in estimating ischemic penumbra^3^ or infarct core^4–6^.

A more efficient method for estimating lesion reversibility utilises NCCT. Acute ischemic lesions become progressively more hypoattenuated on NCCT with time, allowing for a ‘tissue clock’^7–9^. The approach – Net Water Uptake (NWU)^7^ – has been validated relative to lesion progression on MRI^10^, and CTP^11^, and predicts lesion growth^12–14^, and outcomes^9,15^. The NWU method for measuring lesion age from NCCT derives a single feature - relative intensity (RI) – that shows a close linear relationship with tissue water content^7^, and therefore ischemic blood-brain barrier breakdown. However, NWU may be confounded by alternative sources of hypointensity e.g. leukoaraiosis; does not account for within-lesion variability, and is insensitive to additional ischemia features, e.g. focal swelling without hypoattenuation^11,16^. Whether more advanced imaging analysis can significantly improve NCCT tissue clock estimation is unknown.

A further issue is NWU’s dependency upon lesion segmentation. In previous studies, additional imaging, manual annotations^7,10^, or sampling a priori regions^17–21^, are used to identify lesions, which are impractical, subjective, sample non-lesion areas, and provide limited brain coverage.

Here, we describe a method by which ischemic lesions on NCCT are analysed to estimate biological age, that leverages machine learning (ML). Advantages of ML are that it screens high-dimensional imaging features for associations with ischemia progression, including those imperceptible to experts^22,23^, and can account for lesion anatomy variability and signal heterogeneity. ML has proven superior to unidimensional approaches for MRI-based ischemic lesion age estimation^24^, and for NCCT-based prediction of edema^25^. ML also enables objective lesion segmentation from NCCT images^26,27^, that can secondarily be analysed for lesion age. However, because segmentation becomes increasingly inaccurate as acute ischemic lesion age lessens^26,28^, an approach is needed to minimize segmentation errors propagating to lesion age estimation^29,30^.

**Fig. 1:**
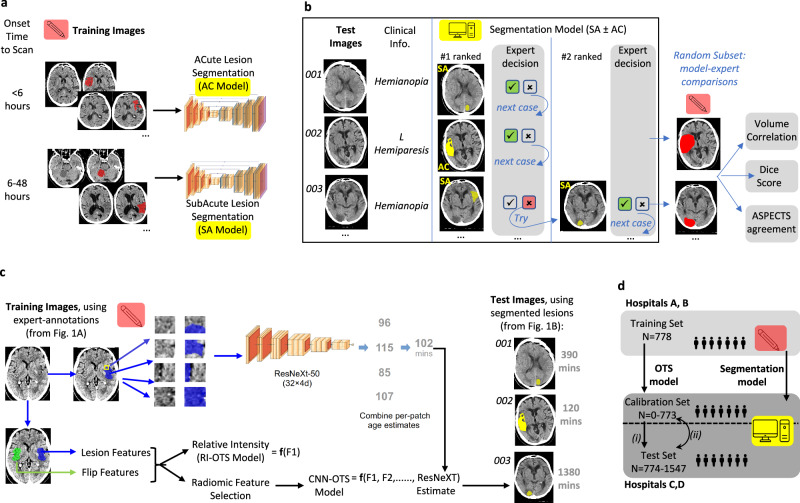
Methods overview. **a** Two ischemic lesion segmentation CNN models were trained using expert-annotated 3D NCCT images: Acute (AC: Onset Time to Scan, OTS: <6 h); and Subacute (SA: OTS: 6–48 h). **b** Validation of lesion segmentations occurred in two ways: (i) experts provided with Test NCCT images and clinical syndromes, judged whether the 1^st^-ranked SubAcute model segmented component overlay the relevant ischemic area (as for Image #001); unless no relevant SubAcute output existed, when the Acute model was judged instead (#002). If the 1^st^-ranked component was incorrect, e.g. lesion unrelated to presentation, or artefact, then the 2nd component was interrogated (#003), and so for the 3rd, but no further components; (ii) a random subsample of segmented, adjudicated components were directly compared with each of two experts’ manual annotations. **c** Lesion age (OTS) model was derived from a CNN generated from multiple patches of expert-annotated ischemic regions (using unregistered NCCT images). The CNN-OTS estimate was combined with radiomic features from the same ischemic region and its flipped control image (using spatially realigned and normalized images). A Relative Intensity (RI) model, equivalent to lesion Net Water Uptake (NWU), was also developed from normalized difference in mean NCCT attenuation within ischemic and flipped masks. The CNN model, supplemented with radiomic features (CNN-R), and RI model, were validated using segmented ischemic lesions from the Test cohort, as per **b**. **d** Model Training, Calibration and Test were performed in three approximately equal number, independent datasets, with Training images derived from different hospitals to those used for Calibration and Test; and with the former using expert-drawn lesions, and the latter using automatically-segmented, adjudicated, lesions. Calibration and Test sets were alternated (i, ii) to generate OTS estimates of all 1547 subjects. Smaller Calibration sets (0–500), and correspondingly larger Test sets, were also evaluated.

Our study took two stages (Fig. 1): firstly, we developed a ML method for automated ischemic lesion segmentation from NCCT, supplemented by expert adjudication. Secondly, appropriately segmented lesions were used to develop a lesion age ML model. This model was trained with chronometric data, leveraging larger, less biased datasets than those where adjunctive imaging is available. We hypothesised that a lesion age ML model would be better at predicting lesion biological age than that predicted by NWU, as well as superior to time itself, given time does not account for reversibility factors such as arterial collaterals^14^.

## Results

Characteristics of the Training and Test cohorts are summarized in Supplementary Table 1.

### Lesion segmentation

**Fig. 2:**
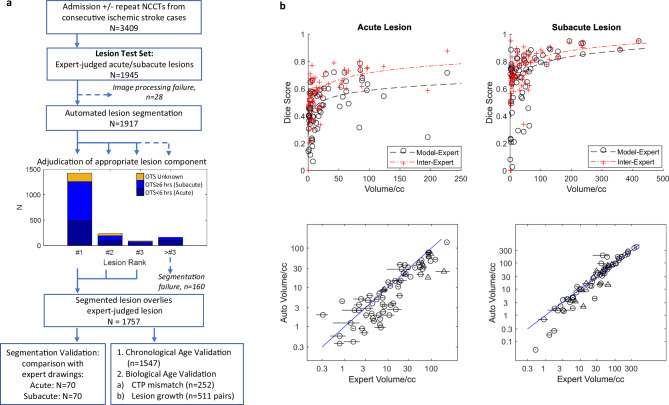
Lesion segmentation validation. **a** Flow chart of Test cohort, showing numbers of expert-adjudicated ischemic lesions identified by either segmentation model, as a function of whether the lesion was identified by the 1^st^, 2^nd^ or 3^rd^ probabilistically-ranked model output component. Lower ranked components were considered segmentation failures. Successfully segmented lesions were then used for validation of i) lesion segmentation spatial similarity (see **b**); and ii) lesion age model compared to chronometric age (OTS), and two measures of lesion biological age. **b** Lesion segmentation fidelity was evaluated in random subsets of 70 acute (OTS < 6 hours: left graphs) and 70 subacute (OTS 6–48 h: right graphs) ischemic lesions identified by one of the top three segmentation model outputs, compared to two expert annotations. Top two graphs show Dice Similarity Coefficients (DSC) of model versus experts, as well as inter-expert comparison, as a function of expert annotation volume, with log function lines of best fit shown. Lower graphs show scatter plots of lesion volume comparing expert annotations versus model (Auto) outputs. Horizontal bars reflect the range of experts. Circles denote thin slice images; triangles denote thick slice images.

The proportion of acute or subacute ischemic lesions identified by either lesion segmentation model was 90.3% (1757/1945; Fig. 2a); with 38% segmented exclusively by the Acute model; and the remainder segmented by the Subacute model. The proportions of lesion-identifying components ranked probabilistically 1^st^, 2^nd^ or 3^rd^ were: 0.81, 0.14 and 0.05 respectively. Agreement for selecting relevant components across 3 experts was 0.90 (45/50; kappa: 0.854).

Similarity metrics of acute and subacute lesion segmentations relative to expert annotations, and inter-expert comparisons, are detailed in Supplementary Table 2 and Fig. 2b. Dice Similarity Scores (DSC) and lesion volume correlation strengths of segmented model outputs versus expert annotations, were smaller than inter-expert comparisons (*p* < 0.05 for all). However, the relative decrease in auto-expert relative to expert-expert segmentation comparisons was only 3–13%. Agreements in ASPECTS did not differ significantly comparing segmentation-expert with inter-expert (comparison of model-expert vs. inter-expert: t(138) < 0.6; *p* > 0.1). DSC increased with lesion age, for both model-expert and inter-expert comparisons: median DSC for 0–9 h: 0.484 and 0.589; versus 9–72 h: 0.745 and 0.779 (*p* = 0.001 comparing time windows for both comparison types). Influences of lesion age, slice thickness and white matter lesion load on segmentation accuracy are further described in Supplemental Material.

### Chronometric age

**Fig. 3:**
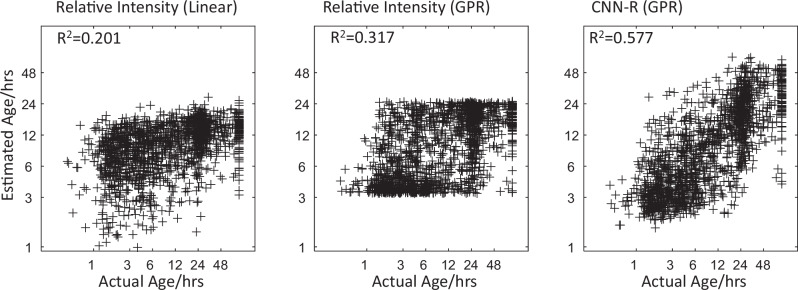
Chronometric OTS validation. Scatterplots of lesion age estimates in Test cohort as a function of actual chronometric OTS. First two models shown use Relative Intensity (Net Water Uptake) alone, either as a linear or a Gaussian Process Regression (GPR) function. Third model uses the CNN model supplemented with radiomic features (CNN-R) developed within the Training cohort. Axes are logarithmic.

Across the entire Test cohort, R^2^ for OTS estimation increased from 0.201 or 0.317 for Relative Intensity model (RI: linear, or Gaussian Process Regression (GPR), models respectively), to R^2^ = 0.577 for CNN-R model (% improvement = 85%; comparison of best RI model vs. CNN-R: F(11,1534) = 86, *p* < 0.001; Table 1; Fig. 3). Splitting the cohort into Thin and Thick images, R^2^ was less for Thick versus Thin images across all models (% reductions of 54%, 25% and 23% for linear NWU, GPR NWU and CNN-R respectively, all *p* < 0.01). However, the relative improvement of CNN-R versus best NWU model was similar for Thin and Thick images (63%, 68%, respectively; difference between CNN-R and NWU: F(11, 383–1138) > 11, *p* < 0.001). Smaller calibration set sizes reduced R^2^ for both models: for *n* = 500, 50, 0, CNN-R: 0.562, 0.407, 0.365; RI: 0.308, 0.260, <0; but the % improvement of CNN-R versus RI remained high (82%, 57%, >100%, respectively; F(11,1535) > 35; *p* < 0.001). Models using only CNN or radiomic features were inferior (R^2^ = 0.370 and 0.507 respectively) to the combined CNN-R model (comparisons: *p* < 0.001).

**Table 1:** Onset time-to-scan estimation: test experiments

	*N*	Relative Intensity (NWU): Linear Model		Relative Intensity: (NWU): GPR Model		CNN-Radiomic: GPR Model	
Performance		R^2^	RMSE (mins)	R^2^	RMSE (mins)	R^2^	RMSE (mins)
All	1547	0.201	1357	0.317	1288	0.577	1063
Thin Slice	1151	0.228	1498	0.360	1377	0.588	1163
Thick Slice	396	0.105	465	0.269	422	0.453	363
		F1 Score	Accuracy	F1 Score	Accuracy	F1 Score	Accuracy
<4.5 h							
All	550	0.319	0.677	0.558	0.719	0.697	0.799
Thin Slice	381	0.314	0.697	0.547	0.730	0.690	0.810
Thick Slice	169	0.364	0.611	0.596	0.674	0.688	0.750
4.5–9 h			0.407* 0.362 0.568		0.467* 0.488 0.636		0.634* 0.632 0.697
All	235	0.254		0.178		0.293	
Thin Slice	158	0.238		0.155		0.280	
Thick Slice	77	0.314		0.209		0.325	
9-24 hrs							
All	248	0.354		0.377		0.456	
Thin Slice	194	0.282		0.271		0.384	
Thick Slice	129	0.250		0.542		0.677	
>24 h							
All	439	0.014	0.718	0.116	0.715	0.634	0.824
Thin Slice	418	0.103	0.652	0.472	0.706	0.691	0.809
Thick Slice	21	0	0.947	0	0.947	0	0.947
Weighted Ave							
All	1547	0.230	0.592	0.337	0.627	0.567	0.746
Thin Slice	1151	0.222	0.578	0.419	0.647	0.583	0.755
Thick Slice	396	0.298	0.607	0.471	0.669	0.577	0.733

R^2^ signifies goodness of fit (coefficient of determination).

*GPR* Gaussian Process Regression.

^a^For accuracy comparisons, the two intermediate time windows were combined to 4.5–24 h in order to create similar proportions of cases across the three windows. Values stated are in order: All, Thin slice, Thick slice.

The influence of time (OTS) on model performance was evaluated in three ways. Firstly, model error magnitudes were regressed against OTS. Both types of RI and CNN-R models showed increased absolute error with time: beta = 584 (552–615), 547 (516–578), 450 (423–476; t(1546) > 33, *p* < 0.001) for linear RI, GPR RI and CNN-R models respectively. However, the regression coefficient for CNN-R was significantly less than those of both RI models (Z = 4.69, *p* < 0.001), indicating that CNN-R’s performance was less time dependent. Secondly, we measured performance of the OTS models at identifying cases within, versus outside, each of four consecutive time windows: ≤4.5, 4.5–9, 9–24, and ≥24 hours (Table 1). A superiority of CNN-R relative to best RI model was seen for all four (McNemar Tests Chi2 > 8.8, *p* < 0.01), with % F1 improvements for the four windows being 25%, 65%, 21%, 445% respectively (and similarly for Thin and Thick images, separately). AUROCs for classification ≤/>4.5 hours were: 0.774 (0.750–0.798) and 0.879 (0.860–0.898) for RI and CNN-R models respectively; and for classification </≥24 h were: 0.762 (0.738–0.787) and 0.895 (0.878–0.913), respectively (model comparisons: Z > 3.3, *p* < 0.001). Thirdly, comparing classification accuracies for time windows <4.5, 4.5–24, and >24 hours, for each OTS model, there were significant decreases for the intermediate window (4.5–24 h) compared to either of the two end windows for each model (Table 1). However, this decrease was significantly less for CNN-R than either RI model (3 ×2 contingency table Chi2 > 796, *p* < 0.001).

There was a weak correlation between lesion segmentation accuracy (corrected DSC) and absolute residuals of the RI model (ρ = −0.184, *p* < 0.05; *n* = 140). Correlations of corrected DSC with CNN-R model residuals, or with differences between the residuals of the two models (absolute or %), were non-significant (ρ >−0.11, *p* < 0.1).

### Biological age: core:penumbra ratio

Subject characteristics for Biological Age experiments are shown in Supplemental Table 3.

**Fig. 4:**
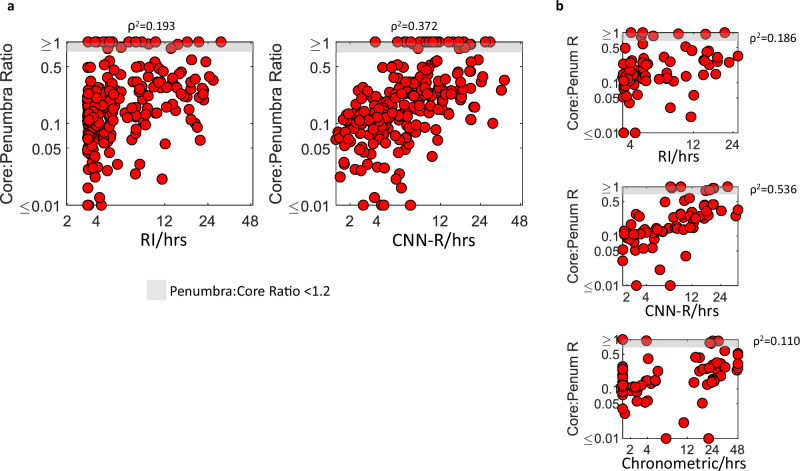
Core:penumbra ratio estimation. **a** Scatterplots of lesion age estimates from paired NCCT-CT Perfusion (CTP) Test subset, comparing against CTP ischemic core : penumbra ratio mismatch. Age estimates originate from the optimal Relative Intensity (GPR) and CNN-R models. Correlation coefficient of plot using CNN-R was greater than that using RI model: Z = 2.62, *p* < 0.01. The grey zone represents cases where penumbra : core ratio is <1.2 (a threshold used to select patients for revascularization). Axes are logarithmic. **b** Scatterplots of a subset of cases where chronometric OTS was also available, showing superiority of CNN-R estimated age relative to both RI model and chronometric OTS (Z > 2.7; *p* < 0.01).

Correlation strengths of core:penumbra ratio (CPR) with OTS estimated by the CNN-R model, applied to the associated segmented NCCT lesion, was 91% greater than OTS estimated by the best (GPR) RI model (ρ^2^ = 0.372 vs. 0.193; Z = 2.62, *p* < 0.01) (Fig. 4a). For a CPR threshold of >0.8 (penumbra:core ratio <1.2), classification of cases based upon estimated OTS was achieved with AUROC of 0.771 (0.710–0.831) vs. 0.663 (0.596–0.731), for CNN-R and RI models respectively (Z = 2.15; *p* < 0.05).

In a subset of 73 subjects, chronometric OTS (cOTS) was available. Correlation strengths of CPR with CNN-R estimated OTS were 3–5× greater than either the best RI model, or cOTS (ρ^2^ = 0.536, 0.186, 0.110, respectively; Z > 2.7; *p* < 0.01; Fig. 4b). For a CPR threshold of >0.8, AUROCs of CNN-R and RI models and cOTS were 0.796 (0.688–0.904), 0.664 (0.538–0.791), and 0.486 (0.370–0.638) respectively (Z = 3.05 for CNN-R vs. cOTS, *p* < 0.01; other two comparisons are non-significant).

### Biological age: infarct expansion

**Fig. 5:**
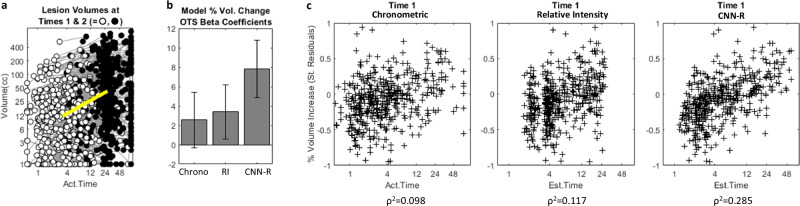
Infarct growth prediction. **a** Scatterplots of ischemic lesion volumes in Validation subset with paired NCCTs at two time points (white and black circles respectively), representing predominantly scans on admission, and 24–48 h follow up. Axes are logarithmic. **b** Comparison of regression coefficients for lesion age (at first scan) as a predictor within a multiregression model of percent ischemic lesion expansion. Lesion age value is taken as either chronometric OTS, or as the estimates from optimized Relative Intensity (RI) or CNN-R models. Only the CNN-R age estimate coefficient compared to the other two age estimates was significant (Z > 2.14; *p* < 0.05). **c** Standardized residuals of a baseline model of percent ischemic lesion expansion plotted as a function of lesion age estimates (at first scan). Negative-y axis values indicate that estimated infarct growth is less than that estimated by a model using baseline predictors of: lesion volume at t1; t2-t1 interval, and stroke severity. The three graphs use chronometric OTS, RI, and CNN-R estimated ages respectively. Correlation coefficient of plot using CNN-R estimated age was significantly greater than plots using the other two age estimates (Z ≥ 3.24; *p* < 0.01).

Figure 5a shows ischemic lesion volume at two time points in 511 NCCT image pairs. The following predictors were independently associated with % infarct expansion: lesion volume at t1 (t(507) = 8.14); t2-t1 (t(507) = 2.17); NIHSS (t(507) = 2.47; all *p* < 0.05); whereas age, sex and thrombolysis treatment were not.

Adding chronometric OTS (cOTS: time from onset to t1) to the above multiregression baseline model, showed a non-significant trend (t(503) = 1.76, *p* = 0.079). By contrast, CNN-R estimated OTS or RI-estimated OTS were both significant (t(503) = 5.49, 2.74 respectively, *p* < 0.01); that remained significant when cOTS was additionally included in the model (t(502) = 5.21 and 2.39 respectively, *p* < 0.05) (Fig. 5b). Comparing regression coefficients, CNN-R OTS was greater than RI-OTS and cOTS (Z > 2.14; *p* < 0.05).

To visualize the influence of OTS, the residuals of the baseline model for % infarct expansion were plotted against the three estimates of OTS (Fig. 5c). This shows greater correlations for CNN-R > RI > cOTS (ρ^2^ = 0.285, 0.117, 0.098). CNN-R OTS r was significantly greater than that using the other two OTS estimates (Z ≥ 3.24; *p* < 0.01), but RI-OTS was not significantly greater than cOTS (Z = 0.521).

## Discussion

We show that a deep learning based biomarker (CNN-R) for estimating acute ischemic lesion age from NCCT is approximately twice as accurate as the current best method, NWU. The reliability and generalizability of our findings is supported by the fact that a similar order of superiority of CNN-R, compared to NWU, models was demonstrated across three independent validation experiments, using separable measures of lesion age: chronometric, core:penumbra mismatch, and as a predictor of lesion growth. Strengths of our study are that the Test images were derived from separate centres to those of the Training set, and used images from multiple scanner types, including image slices 7x thicker. The proportional advantage comparing CNN-R to RI models was seen equally with thick versus thin NCCT images; and when small or no calibration sets were used - indicating that the CNN-R model is robust to variations in NCCT scanners, image quality, and populations.

Whilst the CNN-R model was trained on chronometric age, it also outperformed time itself in predicting lesion biological age. This implies that the CNN-R model tracks ischemic tissue evolution; and mirrors the finding that a chronometric-trained, whole “brain age” CNN-R model predicts age-related disease more accurately than age itself^31^.

Previous studies utilizing NCCT NWU for lesion age estimation report high accuracies for age classification^7,8,10^. Yet, when comparing studies, it is important to consider population distribution, and case mix. Earlier studies typically use smaller, more selective, homogeneous cohorts, often limited to large-vessel, middle cerebral artery (MCA) infarcts, and/or use imbalanced distributions, or early time windows^7,8,10^. In our study, we tested a retrospective consecutive series of ischemic strokes, agnostic of size or anatomy, that is more relevant for clinical application, whilst enriching test data with challenging cases that have intermediate OTS values. Approximately half of cases were >12 h, yet the relationship between an imaging biomarker tissue clock and chronometric age becomes weaker with time^8^, that we found for all models by regressing age prediction error against time. However, the CNN-R model not only remained consistently superior to NWU comparing F1 scores across all four time windows, but was less time dependent, as evidenced by both a smaller regression coefficient of error against time, and smaller differences in accuracy across time windows.

A further advantage over many previous NWU lesion age studies is that our method entails ischemic lesions being segmented from admission NCCT automatically, without further imaging. Earlier studies delineated lesions by cross-referencing CTP or follow-up NCCTs^7,8,10,19^, which limit their practicality. Whilst automated segmentation introduces inaccuracies, the impact of these on OTS estimation was likely small. This is suggested by the facts that segmented lesion extent (ASPECTS) comparisons with experts did not differ significantly from interrater comparisons; and there were no significant correlations between segmentation-expert DSCs versus CNN-R model residuals. We also found that segmentation-expert correlations and DSCs decrease as lesions become more acute, whereas the effect of time on age prediction performance is in the opposite direction (i.e. model error increases with time), implying that lesion segmentation is not the principal limiting factor for age prediction. Furthermore, the fact that CNN-R retains a similar order of superiority over NWU with both early and late lesions – despite lower segmentation accuracies in earlier time windows, shows that the performance advantage of CNN-R is not impacted by less accurate lesion delineations, at least within the range achieved by our method.

The main limitation of our technique is that experts were required to select lesion segmentation model outputs prior to lesion age estimation. However, using a semi-automated, as opposed to fully automated, method was preferable so as to: 1) ensure that in cases with >1 lesion, the appropriate lesion for the presenting clinical syndrome was selected; and 2) minimise errors propagating from lesion segmentation to OTS estimation^29,30^. In ~25% Test images, lesions were not captured by the 1^st^ ranked component, and so would have provided erroneous inputs to a lesion age model, had full automation been used. Commercial, fully automated NCCT ischemic lesion segmentation algorithms carry a ~30% error detection rate^28^; and a CNN-transformer model which combines fully automated lesion segmentation with lesion age estimation^30^ showed less robust results than in the current study. The advantage of our approach can be appreciated by comparing our DSCs for acute and subacute lesions (0.49 and 0.74) versus those reported for fully automated methods^26,27,30^ for similar maturity lesions (0.37–0.45 and 0.61 respectively), noting also that these earlier studies typically used homogeneous cohorts, and so DSCs would likely be lower still in our highly mixed sample. Furthermore, our method allows for more precise and comprehensive sampling of lesions than “automated NWU” techniques which sample NWU across a priori regions of interest^17–21,32^.

The reproducibility and feasibility of our approach is favourable, given a high interrater agreement, and NCCT interrogation taking ~1 min/scan. It is likely that any stroke lesion analytic tool will require expert input because any segmentation method will result in false positives, and often >1 lesion exists^11^, requiring additional information for selection. A further limitation is that our described pipeline cannot age ischemic lesions invisible on NCCT. However, this is unlikely to be of significant consequence because ischemic lesions invisible on NCCT either represent very early ischemia (in 77 cases where subtle lesions were only identified retrospectively, median OTS was 2.4 h); or very small lesions, suggested by a small clinical deficit, for which risky treatments (thrombolysis/thrombectomy) are often not recommended, and MRI is required to establish diagnosis. Conversely, the primary application of NCCT lesion age models is for medium-large strokes which show some ischemic changes – in which the potential for reversibility is unknown, yet it is upon this information which decisions regarding thrombolysis and thrombectomy rest.

We caution that our results may be model-dependent, despite our attempts to verify generalizability across study centre and scan quality. Future models using more advanced deep learning models e.g. transformer or diffusion-based, which sample images across a wider field of view, may achieve superior accuracies and be more generalizable.

The practical importance of our results lies in the CNN-R lesion age biomarker providing more accurate estimates, compared to current methods, of stroke onset time (unknown in ~20% cases), and lesion reversibility, both currently used for decisions regarding revascularization treatments. One of our standards for lesion reversibility was CTP core:penumbra mismatch that is only weakly related to chronometric age^33^ but strongly related to outcome prediction^9^. However, CTP is vulnerable to confounds^6^, and shows inconsistent associations with lesion reversibility^34^, potentially explaining why lesion NWU has been found to be superior to CTP mismatch at predicting outcome^20^.

While it may be considered trivial that a deep learning imaging analysis should outperform a simple thresholding approach such as NWU, there is a priori ground for expecting NWU to suffice in affording an accurate marker of tissue ischemia. This follows from the fact that CT hypoattenuation shows a close linear relationship with tissue water uptake, and ischemia^7^. Regional differences in this relationship are correctable by normalizing with respect to tissue attenuation in the opposite half of the brain. Thus, additional imaging features might be expected to offer no further informative value. However, the approximately two-fold magnitude of the improvement seen with our CNN-R model indicates otherwise, and may be driven by overcoming limitations of the NWU method, such as: hypoattenuation confounding (e.g. chronic white matter lesions, partial CSF voluming, anatomical asymmetry); ignoring signal distribution within a lesion; and insensitivity to additional features that may indicate lesion reversibility, such as focal swelling without hypoattenuation^11^, or collaterals^35,36^. These features may be detectable on NCCT using CNNs even when imperceptible to humans^22,23^, and may explain why a CNN-R model of lesion age allows for higher accuracy than NWU at estimating core:penumbra ratio and lesion growth. Lesion age models using either CNN or radiomic features alone did not achieve the same level of accuracy as a CNN-R model.

In conclusion, our study shows that that a CNN-radiomics based model of ischemic lesion age significantly outperforms the NWU approach. As well as validating the method in a large, independent cohort, we demonstrate that the technique can be embedded within a practical pipeline of automated lesion segmentation and clinically-based expert selection. Future research should assess whether the higher accuracy of a CNN-R approach to lesion age estimation carries over to predicting lesion reversibility and functional outcomes.

## Methods

### Dataset

The Training set comprised 783 volumetric NCCT scans from 665 retrospective acute ischemic stroke patients presenting to two hospitals, where time between symptom onset and imaging (Onset Time to Scan, OTS) was known; and ischemic lesions were identifiable on NCCT to a neuroradiologist. Lesions were divided into acute (OTS < 6 h) and subacute (OTS ≥ 6 h). An independent Test set comprised 3409 volumetric NCCT scans, from 2502 retrospective ischemic stroke cases, presenting to two additional hospitals. From these 3049 cases, 1945 scans were identified with visible acute or subacute lesions. In 1509 Test cases these represented consecutive presentation +/− first follow-up images (where available). No subjects provided scans to both Training and Test sets. However, since OTS distribution from the consecutive series was bimodal (peaks: ~3 and 25 h), the Test lesion set was enriched with a further 436 images from subsequent consecutive cases originating from the same Test hospitals, where scans took place with an intermediate OTS (4.5 < OTS < 18 h). Histograms of OTS distributions in Training and Test sets are shown in Supplementary Fig. 1. Training set scans were exclusively Thin-sliced (median: 0.45 × 0.45 × 1.0 mm), whereas the Test set consisted of 72% Thin, and 28% Thick sliced scans (2.4 mm base, 7.2 mm cerebrum), allowing us to assess model transference to NCCTs of different image quality. In 77/1757 cases (4.4%) where ischemic lesions were appropriately segmented (see below), the original hospital NCCT radiology report did not observe an ischemic lesion, either of because of human error, or because an area of equivocal hypoattenuation was later judged to represent acute ischemia by an expert based upon judging a subsequent NCCT or MRI. Median OTS for these cases was 2.38 h (IQR:1.61–4.74).

Ethical approval was granted by the UK Health Research Authority Research Ethics Committee (Centre: Wales: Reference16:/WA/0361). Informed consent was waived by the ethics committee as all utilised data was retrospective, acquired for the sole purpose of clinical care, and anonymised, whilst the study aims were judged to be beneficial for future patient care.

### Lesion segmentation and validation

Since multi-class CNNs for NCCT ischemic lesion segmentation, grouped by lesion intensity/age, are more accurate than single-class CNN^26^, two 2.5D U-Net models^37^ were developed, trained with either Acute (OTS < 6 h) or Subacute (OTS 6–72 h) images. Each utilized a stack of 15 consecutive slices with an in-plane resolution of 256 ×256; four level architecture, incorporating standard blocks comprising convolutional, pooling, and unpooling layers, along with batch normalisation layers. During training, the model underwent 100 epochs with a batch size of 128. The Adam optimiser^38^ with a learning rate of 0.0001 and cross-entropy loss was used. Ground truth acute / subacute lesion segmentation masks in 79,959 NCCT slices were produced by manual annotations from one of three board-certified stroke imaging experts with >10 years experience each. Inter-expert agreement of ischemic lesion size estimation (ASPECTS score) for 24 acute lesions was good: Cohen’s kappa: 0.74–0.79. NCCT images were pre-processed using a recommended pipeline^39^. Pixel intensities were clipped at 0.5 and 99.5 percentiles, and normalized using Z-score.

Models generated a probabilistic volume, from which the three highest ranked, spatially-connected components were extracted (i.e. whose maxima represented the three highest probabilities), thresholding at *p* > 0.5. Three was chosen as a practical number for rapid expert evaluation, and because of diminishing returns for components beyond the third (Fig. 2a). Both models were run for each image. If the Subacute lesion model identified the relevant lesion, then this was carried forward to lesion age estimation; whereas if the Subacute lesion model did not identify the relevant lesion, then the Acute lesion model output was used. The rationale for this is that the Subacute lesion mask is more likely to capture lesion subparts affected from stroke *onset*; and to be more accurate, than acute lesion segmentations^26^; but the Acute lesion model is more sensitive to subtle areas of tissue hypoattenuation.

Segmentation validation occurred as follows (Fig. 1b): Firstly, we determined the proportion of Test images identified by the lesion segmentation models. Experts adjudicated whether the 1^st^-ranked segmented lesion component overlay the relevant ischemic lesion, given concise clinical information e.g. hemiparesis side (Fig. 1a). If the model output was judged as incorrect, e.g. artefact, or incorrect lesion-syndrome pairing, then the 2^nd^-ranked component was assessed, and similarly for the 3^rd^. If none of the top three components identified the relevant lesion, this was termed a segmentation failure. Adjudication was performed by one of three experts; with 50 cases adjudicated by all to ascertain interrater reliability of lesion identification. A graphical-user interface enabled efficient adjudication (median: 75 s per case).

Secondly, we assessed for spatial similarity between segmented and actual lesions. Random subsamples of NCCT images with acute (*n* = 70) or subacute (*n* = 70) lesions, from which the relevant component had been segmented, were selected. Two experts independently annotated these images, from which the following metrics were determined: Dice Similarity Coefficient (DSC); volume correlation; and Cohen’s Kappa of spatial extent (Alberta Stroke Programme Early CT Score, or ASPECTS); these being assessed between expert masks and segmented components; and between experts themselves. DSC was modelled as log functions of lesion volume, from which corrected DSC values (=actual − estimated DSC) were calculated, to explore associations between lesion segmentation accuracy and OTS estimation. We also assessed effects on lesion segmentation accuracy of lesion age (OTS), image slice thickness, and chronic white matter lesion volume using an automated segmentation algorithm^40^.

### Chronometric age model

A lesion age model was developed from a convolutional neural network based upon 3D cubes of segmented NCCT lesion images, supplemented with radiomic features (CNN-R model).

OTS was log-transformed to account for skewed distribution. Images were linearly interpolated to an isotropic spatial resolution of 0.449 mm^3^. From each appropriately segmented lesion mask, we randomly sampled multiple cubes (each 48 × 48 × 48 voxels, corresponding to 21.6 × 21.6 × 21.6 mm³) centred on random points within the mask. These cubes could extend beyond the mask boundaries. An ensemble of 3D ResNeXt-50-32x4d^41^ models were trained using a 5-fold cross-validation approach. Training consisted of 3000 epochs with a batch size of 48 and the Adam optimiser was used with a learning rate of 0.001 and mean absolute error as the loss function. At Test, predictions are averaged for 1000 randomly sampled lesion-containing cubes, each providing its own age prediction.

To improve estimation of lesion age we combined CNN-estimated value with radiomic features, derived from the lesion +/− its mirror image in contralateral brain^42^. For this step NCCT images were realigned and normalized^43^ and thresholded to exclude bone, CSF and old infarcts. In the Training Phase, 250 radiomic features were extracted by sampling standard statistical metrics of the lesion itself, or normalized difference relative to contralateral brain; of lesion as a whole, or from those weighted by lesion depth; and from spatial, shape and texture features derived using two open source toolboxes: https://github.com/mvallieres/radiomics/ and https://uk.mathworks.com/matlabcentral/fileexchange/25261-lacunarity-of-a-binary-image/^44^. Feature reduction entailed filtering variables with low variance and high cross-correlation, before using stepwise forward feature selection, bootstrap resampling, and GPR regression with R2 as criterion^44^, using only Training set images The following features were selected:

Lesion only features: intensity; skew; depth-weighted median; depth-weighted interquartile range; depth-weighted skew; Gray-Level Co-occurence Matrix correlation.

Difference between lesion and contralateral image features: volume of thresholded minimum; depth-weighted standard deviation; depth-weighted kurtosis; Neighbourhood Gray-Tone Difference Matrix Busyness.

At Training, lesions were delineated by experts; and at Test, by automated segmentations and expert selection. The purpose of lesion selection was to ensure that the lesion was relevant to the stroke presentation from which OTS was based on; and to minimise segmentation errors^26,28,30^ propagating to lesion OTS estimation.

At Test, the CNN-R OTS estimate, plus relevant radiomic features identified from the Training set, were combined within a single Gaussian Process Regression (GPR) model. The Test set was split into two: one half of which calibrated the model; the other then used for test; and vice versa. Coefficients of determination (R^2^) averaged over 20 random splits are reported. Effects of smaller calibration set sizes (0–500) were also assessed.

For comparison, a Relative Intensity (RI) model. equivalent to NWU^7^ was generated, as a single predictive feature, calculated as: mean attenuation of NCCT voxels within the lesion mirror image (“normal side”), minus those within the lesion mask, divided by the mirror image attenuation. Only voxels within an intensity range of 20–80 HU were sampled. RI models evaluated at Test used linear regression, or GPR, the latter of which optimized R^2^ during cross-validation within the Training set.

### Biological age validation – CT perfusion

Lesion biological age was firstly estimated as the mismatch ratio of core versus penumbra volumes, defined respectively as relative hemispheric cerebral blood flow decrease of <30%, and Tmax >6s^45^, measured from paired CTP. A total of 315 acute CTP images were available from Test set subjects. Of these, 252 CTP images showed both a visible ischemic lesion on contemporaneous NCCT (segmented as above), and an overlapping core and/or penumbra region. Relevant NCCT lesions were sampled using the above OTS models, and their estimates correlated against mismatch ratio.

CT perfusion images were acquired from Siemens Somatom 128 slice CT angiography, using the following parameters: acquisition resolution 128 ×0.6 mm, slice width: 1 mm, pitch: 0.55, kV: 120; mAs: CARE dose; SAFIRE level: 3, DRL: 800. CT-Perfusion maps were derived from 4D angiography data using an established approach^46^: 1) Motion was corrected by registering all time frames to the first time frame^47^; 2) Spatial noise reduction using an edge-preserving anisotropic diffusion filter with a gradient modulus threshold of 30, an integration constant of 0.1, and a maximum of 50 iterations^48^; 3) Time frames were linearly interpolated to ensure a continuous rate of 1 per second; 4) The skull was stripped using the brain extraction tool from FMRIB Software Library (FSL)^49^ with a fractional intensity of 0.1^50^; 5) Correction of haematocrit differences by applying a scaling factor of 0.73^51,52^; 6) Correction of partial volume effects in the arterial input function (AIF) by rescaling with the area under the curve of the venous outflow function (VOF)^53^. The AIF and VOF were measured using a robust algorithm^54^, and outputs visually verified to resemble physiological attenuation patterns before further processing; 7) Computation of perfusion parameter maps were performed using singular value decomposition with a block-circulant deconvolution matrix^55^ that allows for tracer arrival timing-insensitive flow estimates^56^. The following maps were computed: cerebral blood flow (CBF), cerebral blood volume (CBV), mean transit time (MTT), and time to maximum (T-Max). Maps were co-registered with their corresponding NCCT by applying a rigid affine transformation that used the first time-frame CT perfusion scan as a template. Neuroradiologists inspected the outputs to ensure that thresholded ischemic areas related to the acute/subacute ischemic region on NCCT.

### Biological age validation – lesion growth

Ischemic lesion age, estimated either as NWU or OTS, are independent predictors of infarct growth over the first ~24 h^13,57^, and so we compared the strength of prediction of NCCT-based lesion age models and chronometric OTS. From the Test cohort, 511 cases were available where two NCCT images were obtained at different time points (t1, t2) and an acute/subacute lesion was visible on the t1 image. These pairs mostly represent scans on admission, and then 1-2 days later. Volumes of the relevant acute/subacute lesion on the t1 and t2 images were determined via segmentation (as above), from which % lesion growth was calculated. Lesion volumes were corrected for edema^5^. However, conclusions do not differ if uncorrected values are used.

Percent lesion growth was regressed against lesion volume at t1; delay between t2 and t1; baseline stroke severity (NIHSS); age; sex, and thrombolysis treatment, to determine a baseline model. Subsequently we determined the effect of adding chronometric, or RI, or CNN-R, model-estimated OTSs to the baseline model, and compared regression coefficients between these predictors.

### Statistical analysis

Continuous variables are presented as medians and interquartile ranges, for non-normal distributions; or means and 95% confidence intervals otherwise. Comparisons of group characteristics used non-parametric tests. Correlation coefficients reported are Spearman type (ρ). Comparisons of R^2^ used F test; of correlation coefficients used Fisher Z transformation; of regression coefficients used Z test; and of areas under receiver operating curves (AUROC) used deLong Z test. Tests were conducted in MatLab (v2022).

## Supplementary information

Supplemental Material - NOT MARKED-UP

## Data Availability

Anonymized data are available from the corresponding author upon reasonable request.

## Code availability

The underlying code for this study are available from the corresponding author upon reasonable request.
