# Supplementary material for: Deep learning biomarker of chronometric and biological ischemic stroke lesion age from unenhanced CT
Source: NPJ Digit Med. 2024 Dec 6;7:338. doi: 10.1038/s41746-024-01325-z (PMC11624201; doi:10.1038/s41746-024-01325-z)

Supplemental Material

Factors influencing Lesion Segmentation

Segmentation success, measured as proportion of NCCTs with acute/subacute ischemic lesions whose lesions were identified by the first segmented spatial component - was influenced by lesion age, and image slice thickness: lesions <6 hours were identified by 1st component in 0.678 (498/734); lesions ≥6 hours: 0.797 (762/956) (Chi2: 36.4, p<0.001). For Thin images (≤1mm), lesions were identified by 1st component in 0.771 (901/1169); Thick images: 0.689 (359/521) (Chi2=26.2, p<0.001). DSC for model-expert comparisons was greater for subacute (lesion age 9-72 hrs) than acute (0-9 hrs) lesions (ranksum p<0.001; Supplementary Table 2) but did not differ between Thin versus Thick-sliced images for both acute and subacute lesions (p>0.1). White matter lesion volume did not significantly correlate with DSC (r > -0.202, p>0.05).

Supplementary Table 1. Cohort characteristics

Cohort	Training		Test			P*
N images	783		3409			
N per hospital	A: 737, B: 46		C:2484 , D:925			
	Acute (<6hrs)	Subacute (≥6hrs)	No visible lesion	Lesion: segmented	Lesion: failed segmentation	
	466	317	1464	1757	188	
N subjects	665		1068	1385	176	
Age	75.0 (63.9-82.9)		74.7 (61.0-84.0)	74.0 (62.0-83.0)	76.0 (60.0-84.3)	n.s.
Females (%)	48		48	50	46	n.s.
Onset Time to Scan / hrs	3.9 (1.80-20.9)		4.4 (2.1-20.5)	9.7 (3.1-25.8)	2.7 (1.7-10.7)	<0.001
Stroke severity / NIHSS (/42)	13 (7-20)		5 (2-10)	11 (5-18)	9 (4-17)	<0.001
Thick sliced (>2mm) (%,n)	0		23.0 (336)	24.8 (436)	53.7 (101)	<0.01
Test Images successfully segmented and used for chronometric age validation						
Slice type			Thin (n=1151)	Thick (n=396)		
Onset Time to Scan / hrs			10.9 (2.9-28.2)	5.3 (2.9-15.8)		<0.001

All cases were diagnosed with ischemic stroke or transient ischemic attack at hospital discharge. Demographic variables are reported as: Median (IQR). *Comparisons of 3 Test subsets were by Kruskal-Wallis test for continuous/ordinal variables, and Chi2 for case frequencies. NIHSS: National Institute of Health Stroke Scale. MCA: Middle Cerebral Artery. ASPECTS: Alberta Stroke Programme Early CT Score (10=no MCA territories; 0=all territories showing ischemic change).

Supplementary Table 2. Lesion segmentation validation experiments

	Acute Lesions		Subacute Lesions	
Total N, (Thick Slice N)	70, (10)		70, (10)	
Age	70.1 (58.9-80.0)		74.8 (65.0-80.5)	
Onset Time to Scan / hrs	3.3 (1.8-4.7)		24.9 (20.5-41.3)	
Stroke severity / NIHSS (/42)	13 (4-19)		14 (10-20)	
Lesion extent / ASPECTS (/10)	6 (4-8)		4 (2-7)	
Prop. of lesions involving MCA Territory	0.943		0.957	
White matter lesion volume (cc)	3.35 (1.46-12.4)		4.69 (1.15-10.0)	
Spatial Similarity (Dice Score)				
Auto vs. Expert 1	0.478 (0.339-0.591)		0.755 (0.620-0.842)	
Auto vs. Expert 2	0.496 (0.371-0.595)		0.723 (0.613-0.820)	
Inter-Expert	0.583 (0.470-0.676)		0.765 (0.668-0.849)	
Thin Slice: Auto vs. Expert average	0.484 (0.391-0.580)		0.745 (0.627-0.839)	
Thin Slice: Inter-Expert	0.563 (0.469-0.662)		0.779 (0.669-0.854)	
Thick Slice: Auto vs. Expert average	0.474 (0.315-0.633)		0.708 (0.329-0.735)	
Thick Slice: Inter-Expert	0.654 (0.471-0.731)		0.732 (0.632-0.809)	
Lesion age (OTS) Window (hrs)	0-4.5	4.5-9	9-24	24-72
Auto vs. Expert (average) Dice Score	0.469 (0.352-0.581)	0.535 (0.402-0.653)	0.738 (0.674-0.826)	0.749 (0.646-0.832)

Inter-Expert Dice Score	0.583 (0.473-0.648)	0.641 (0.444-0.723)	0.775 (0.668-0.812)	0.783 (0.697-0.870)
Lesion Volume (Spearman's ρ^2)				
Auto vs. Expert average	0.775		0.907	
Inter-Expert	0.927		0.956	
Lesion Extent, ASPECTS (Cohen's kappa)				
Auto vs. Expert 1	0.662 [0.440-0.884]		0.811 [0.651-0.971]	
Auto vs. Expert 2	0.685 [0.470-0.900]		0.809 [0.649-0.969]	
Inter-Expert	0.744 [0.550-0.938]		0.849 [0.703-0.995]	
Prop. with ≤1 point discrepancy				
Auto vs. Expert average	0.705		0.829	
Inter-Expert	0.786		0.886	

Median (IQR) or [95% CIs]. *Images had either 1mm slices, or 3mm (Thick) slices.

Supplementary Table 3. Characteristics of Test Cohorts for two measures of biological age.

	CTP Mismatch	Lesion Growth
N	252	504
Female (%)	51	51
Age	72 (63 – 81)	71 (61-82)
NIHSS	11 (7 – 17)	12 (6-19)
Acute revascularization therapy / N (%)	n/a	244 (48)
Penumbra Volume / ml	54 (10 – 121)	n/a
Core Volume / ml	8 (1 – 19)	n/a
OTS: 1st UECT / hrs	4 (2 – 22) [n=73]	3.3 (1.6-7.1)
OTS: 2nd UECT / hrs		27 (12-50)

Supplementary Figure 1. Histograms of Training and Test sets. Distribution of cases as a function of OTS. Both sets show bimodal distributions centred around 2.5 hours and 24 hours, although the Test set was enriched with intermediate OTS value cases.

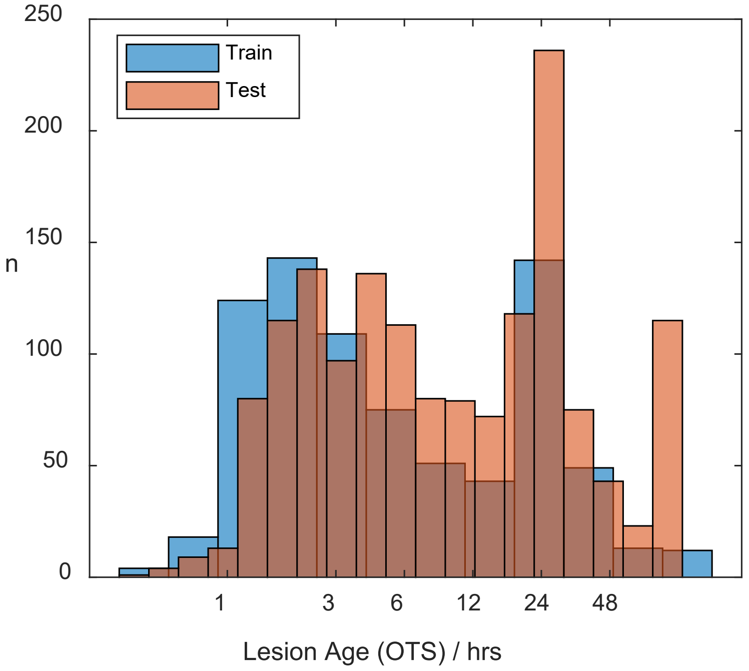

Supplement: Supplementary file 1 — Supplemental Material - NOT MARKED-UP [file 41746_2024_1325_MOESM1_ESM.pdf]
